# Biologics for Chronic Rhinosinusitis—A Modern Option for Therapy

**DOI:** 10.3390/life13112165

**Published:** 2023-11-05

**Authors:** Romica Cergan, Ovidiu Nicolae Berghi, Mihai Dumitru, Daniela Vrinceanu, Felicia Manole, Crenguta Sorina Serboiu

**Affiliations:** 1 Anatomy Department, Carol Davila University of Medicine and Pharmacy, 020021 Bucharest, Romania; r.cergan@gmail.com; 2 Saint Mary Laboratories and Clinics, Carol Davila University of Medicine and Pharmacy, 011013 Bucharest, Romania; oberghi@yahoo.com; 3 ENT Department, Carol Davila University of Medicine and Pharmacy, 050472 Bucharest, Romania; 4 ENT Department, Faculty of Medicine, University of Oradea, 410073 Oradea, Romania; manole.felicia@gmail.com; 5 Cellular Biology and Histology Department, Carol Davila University of Medicine and Pharmacy, 020021 Bucharest, Romania; crengutas@yahoo.com

**Keywords:** biologics, chronic rhinosinusitis, ENT

## Abstract

Chronic rhinosinusitis (CRS) is an important ENT pathology which affects about 5–12% of the general population. The treatment of CRS can be pharmacological (nasal sprays, douches, systemic antibiotics and steroids), surgical (endoscopic sinus surgery) or immunological according to established algorithms. CRS was divided for many years into CRS with nasal polyps (CRSwNP) and CRS without nasal polyps (CRSsNP). New ways of classifying CRS by endotypes (presence of neutrophilia, eosinophilia, fibrosis, glandular hypertrophy and epithelial dysmorphisms) appeared after the most recent understandings of the pathophysiology of the disease. Other classifications divide CRS into primary and secondary forms, localized/diffuse types and anatomical presentation. A new type of treatment has been administered in the last years, biologics. For the moment, biologics are indicated just in the cases of the patients who have undergone ESS or have contraindications for surgery and have bilateral polyps and meet a minimum of three of the following criteria: the necessity for systemic therapies with oral or parenteral corticosteroids or contraindications to systemic steroids, significant loss of smell or impaired QoL score, comorbid asthma and type 2 inflammation. This article aims to present the most relevant studies which used the three types of biologics (anti-IgE, anti-IL5 and anti-IL4/IL3) and wishes to increase the awareness of this new type of treatment that can be used in some CRS cases.

## 1. Introduction

Chronic rhinosinusitis (CRS) is defined as an inflammatory disease of the mucosa of nose and sinuses that lasts for more than 12 weeks without resolution of symptoms and signs. CRS is estimated to affect about 5–12% of the general population. CRS was classified by ENT specialists as CRS with nasal polyps (CRSwNP) or CRS without nasal polyps (CRSsNP). CRSwNP can be classified into eosinophilic CRSwNP (eosCRSwNP) or non-eosinophilic CRSwNP (non-eosCRSwNP) based on the extent of eosinophilic inflammation in the polyp tissues [1,2]. The diagnosis of CRS implicates medical history, physical examination with nasal endoscopy and, if necessary, imaging features of mucosal inflammation. The treatment of CRS includes pharmacological therapy with nasal sprays, douches, systemic antibiotics, steroids, immunomodulators and surgical intervention such as endoscopic sinus surgery (ESS). Biologics were used in the last years with success in order to reduce the need for surgery and increase the quality of life of patients.

## 2. Pathophysiology

The mechanisms implicated in CRS pathogenesis reveal a complex interplay between immunity, airway epithelium, genetics and environmental factors [2]. External factors (bacteria, viruses, fungi) and environmental factors (air pollution, dust, allergens) will activate TLRs (Toll-like receptors), epithelial proinflammatory cytokines production, epithelial defense molecules production, mucociliary clearance and solitary chemosensory cells activation. These factors will activate innate immune cells and adaptative immune cells. Chronic inflammation is the result of this activity with apparition of tissue remodeling, goblet cell hyperplasia, tight junctions disruption, activation of solitary chemosensory cells and bacteria dysbiosis. The clinical picture based on these modifications will translate into elements like nasal congestion and discharge, facial pressure or pain, and reduction in or loss of smell [2].

Chronic diseases have been classified by genotype, phenotype and/or endotype in order to understand the observed variability in the clinical picture and outcomes. Endotypes classification subdivides CRS based on pathobiological mechanisms such as the presence of an excess of neutrophils, eosinophils, elements of fibrosis, glandular hypertrophy and epithelial dysmorphisms. One way to endotype CRS is to use molecules produced by the T lymphocytes of types 1, 2 and 3 that produce the primary cytokines that drive the inflammatory patterns discovered in the tissues. T1 endotype is characterized by the preferential expression of IFN-γ, produced from Th1 cells, cytotoxic T cells, NK cells and group 1 innate lymphoid cells (ILC1s); T2 endotype is characterized by the IL-4, IL-5 and IL-13 produced from Th2 cells, mast cells and ILC2s and eosinophils; IL-17A and IL17F are produced from Th17 cells and ILC3 which are characteristic of T3 endotype. In addition to these leading markers, a number of other biomarkers belong to these endotypes: T1 (CXCL9, CXCL11, GZMH, ZNF683, FCRL6, SLCO1B3), T2 (EPX, CCL18, CCL26, CCR3, CST1, CST2, CLCA1, FCER2, POSTIN, PTGDR2, SIGLEC8) and T3 (IL1B, IL8, CXCL1, CXCL2, CXCL6, CCL20, CHI3L1, SAA1, SAA2, NOX1). Patients may fit in one of these endotypes or can present mixed mechanisms T1,2, T1,3 and T2,3. Few patients are T untypeable (Tun), which patients do not express elevated levels of any kind of biomarker genes, or the T1,2,3 endotype comprises a group of patients that have elevated levels of all three sets of biomarker genes with none of them being the most relevant [3]. Type 2 endotype is predominantly displayed in patients with CRSwNP in Western countries, whereas a mixed type 2 endotype is predominantly displayed on the Asian continent [4]. In addition to distinct immunological mechanisms between Asian and Western CRSwNP patients, different bacterial colonization was noticed [5]. Another way to endotype CRS is to use histopathology: paucigranulocytic, eosinophilic, neutrophilic and mixed granulocytic [6]. Grayson et al. proposed some years ago a simple and practical system of classification. They suggested that the functional anatomical compartments involved in CRS should be a diagnostic tool for the first level of separation into local and diffuse CRS, which can be unilateral or bilateral in distribution. “Pansinusitis” implies that diffuse means the disease is not confined to a known functional anatomical unit. Local anatomical factors are associated with pathogenesis in this classification [7]. Phenotypic classification is determined from the presence or absence of nasal polyps, and comorbidities define the phenotypic classification [8]. Epithelial cells play an important role, being an active component of the immune system of the nasal area and sinuses. They are crucial in the initiation and regulation of immune responses, playing roles in the prevention, development and progression of CRS by actively communicating with immune cells and orchestrating immune responses [9]. A team of experts in the field conducted multiomic single-cell RNA sequencing (sc-RNAseq) of nasal turbinate (control) and sinus tissue. Epithelial and mesenchymal cells were shifted to inflammatory cells in patients with uncontrolled severe chronic rhinosinusitis with nasal polyp compared to healthy nasal mucosa of control people. The expansion of CD4+ Tem and B/Plasma cells was consistent with the elevation of type 2 cytokines and immunoglobulins in NPs [10]. Non-type 2 inflammatory endotypes in chronic rhinosinusitis were proposed in patients with CRS. The mechanisms driving the pathogenesis of non-type 2 endotype in CRS are currently unknown. This varies with race, geography, environment and lifestyle and is more common in Asian patients [11]. The endotyping of patients with CRS according to inflammatory and remodeling factors was to classify patients. Luminex, ELISA and ImmunoCAP were used to analyze forty-eight inflammatory and remodeling factors in the nasal mucosal tissues of 128 CRS patients and 24 control subjects from northern China. Five clusters resulted: clusters 1 and 2 showed non-type 2 signatures with low biomarker concentrations; cluster 3 involved a low type 2 endotype with the highest expression of neutrophil factors (granulocyte colony-stimulating factor, IL-8, myeloperoxidase) and remodeling factors (matrix metalloproteinases, fibronectin); cluster 4 exhibited moderate type 2 inflammation; cluster 5 exhibited high type 2 inflammation with relatively higher levels of neutrophil and remodeling factors [12].

## 3. Biologics Used in the Field of Rhinology

**Figure 1:**
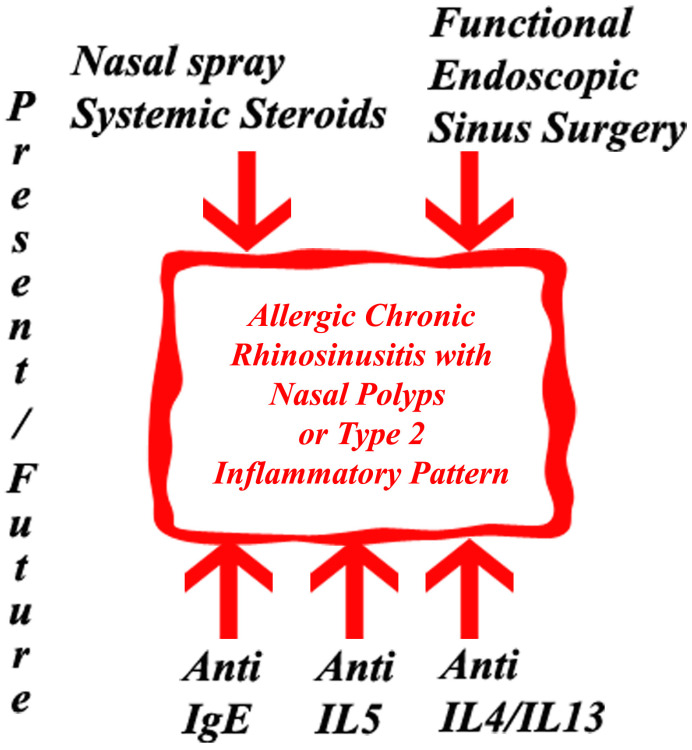
Present and future treatment options in allergic chronic rhinosinusitis with nasal polyps or type 2 inflammatory pattern.

Monoclonal antibodies (MAbs) are biologics used in disorders where immune system dysfunctions are noticed like asthma, psoriasis and atopic dermatitis. Common Th-2 immune pathway is the reason for most of the biologics developed for asthma being effective in CRSwNP. Biologic treatment can be used in CRSwNP patients meeting the eligibility criteria [13]. EPOS 2020 has made indications for the biological treatment of CRSsNP and established the criteria used for the evaluation of the efficiency assessment of biologic therapy. Currently, patients who underwent endoscopic sinus surgery or have contraindications for surgery receive biologic treatment if they meet a minimum of three of the following criteria: a need for systemic corticosteroids or contraindications to systemic steroids, significantly impaired quality of life, hyposmia, comorbid asthma and type 2 inflammation. Reduced NP size, a reduced need for systemic corticosteroids, improved QoL, improved sense of smell and reduced impact of corticosteroids are the criteria used to evaluate the efficiency of biologic therapy [14]. Otherwise, the patients are captive to a combination of topical and systemic steroid regiments and recurrent functional endoscopic sinus surgery, Figure 1.

### 3.1. Anti-IgE

IgE is an antibody that is produced by specialized cells and is involved in a number of human pathologies: allergies, eosinophilic or parasitic diseases. A growing body of evidence shows that IgE may have an important role in the diagnosis and prognostication of CRS [1]. Total serum IgE was observed to present significantly higher levels in recurrent CRS patients when comparing with controls [15]. Tissue IgE concentrations were increased in eosCRS polyps as compared to controls in a Japanese study [16]. High nasal IgE as well as high nasal IL-5 levels were specific to CRSwNP in a study involving 38 patients with rhinosinusitis and controls conducted by Riechelmann [17]. Omalizumab is a recombinant DNA-derived humanized (IgG1k) monoclonal antibody that connects to free human immunoglobulin E (IgE) in the blood and interstitial fluid, and to the membrane-bound form of IgE (mIgE) present on the surface of cells with mIgE. The main effect is reducing the levels of IgE in serum and tissues, with a later blocking of the IgE-mediated inflammatory cascade [18]. Omalizumab reduced peripheral blood eosinophil counts, nasal polyps, sinus opacification with eosinophil-dominant infiltration, FcεRI expression on circulating dendritic cells, airway infiltration of CD4+ T cells, eosinophils, FcεRI expression cells and lower CCL4 release [19]. Rui Zu, based on POLYP1 and POLYP2 studies, concluded that free IgE suppression post-treatment was within the target range of the baseline IgE- and body weight-based omalizumab dosing table in patients with CRSwNP similar to asthma patients [20]. Subjects with CRS despite treatment (including surgery) were included to receive omalizumab or placebo for 6 months in a randomized, double-blind, placebo-controlled clinical trial in an American study. Subjects in the omalizumab group showed signs of reduced inflammation on imagistic evaluation after treatment, whereas those in the placebo group showed no change. Treatment with omalizumab was associated with improvements in the Sino-Nasal Outcome Test at 3, 5 and 6 months of therapy compared to baseline and without important changes in the control group. All the other variables studied presented no important differences between patients and controls [21]. Omalizumab was administered to six patients with a subtype of chronic rhinosinusitis with nasal polyps enriched with eosinophilic rhinosinusitis–eosinophilic chronic rhinosinusitis. This study demonstrated that omalizumab improved rhinological symptoms (SNOT-20, scores for nasal blockage and disosmia) and sinus CT scores (changes in closing volume) and controlled asthma in ECRS patients with severe asthma [22].

Two phase 3, identical, randomized, multicenter, double-blind, placebo-controlled studies (POLYP 1 and POLYP 2) evaluated the efficacy and safety of omalizumab in patients with inadequately controlled CRSwNP despite daily INCS therapy. The studies were conducted across 82 ENT clinics in North America and Europe between November 2017 and March 2019 (POLYP 1) and November 2017 and 7 March 2019 (POLYP 2). Patients aged 18–75 years with persistent bilateral nasal polyps, nasal congestion and impaired HRQoL were randomized. POLYP1 randomized 138 patients and POLYP2 randomized 127 patients. Smell, postnasal drip, runny nose and University of Pennsylvania Smell Identification Test score were significantly improved for the omalizumab group versus the placebo group at week 24—NPS, nasal congestion score and SNOT-22. Endoscopic, clinical and patient-reported outcomes in severe CRSwNP with inadequate response to intranasal corticosteroids were significantly improved by omalizumab that was well tolerated [23]. An open-label extension study continued the initial studies to achieve a total of 52 weeks of treatment. Patients who continued omalizumab reported improved end points. Favorable responses across end points through week 52 were experienced by patients who continued omalizumab. After patients stopped omalizumab, the scores gradually decreased over the 4 months of follow-up but remained better than pretreatment levels [24]. A recent study from 2022 examined the efficacy of omalizumab versus placebo in patients with CRSwNP from the replicate POLYP 1 and POLYP 2 trials, based on individual characteristics, to determine the response to therapy. A broad efficacy of omalizumab across clinical and patient-reported outcomes in patients with CRSwNP, independent of the underlying factors examined, including high eosinophil levels and a history of previous surgery, which are associated with high recurrence, was suggested by the data obtained in the study [25]. Monoclonal antibodies dupilumab (in 49 patients) and omalizumab (21 patients) showed effectiveness in a real-world study in the treatment of severe CRSwNP. Olfactory function, quality of parameters and nasal polyp scores were improved after three months [26]. A rapid improvement of SNOT-22 and ACQ-7 scores were recorded after 4 and 16 weeks in patients with concomitant CRSwNP and asthma after treatment with omalizumab [27]. Patients with recalcitrant CRSwNP and mild asthma have benefited from treatment with omalizumab based on NP size and SNOT-22 outcomes [28]. Omalizumab proved to be an effective treatment in 17 patients with CRSwNP with or without concomitant asthma. Sinonasal outcome parameters significantly improved: SNOT-22, NOSE score and VAS [29]. The beneficial effectiveness of omalizumab was demonstrated in Chinese patients with difficult-to-treat chronic rhinosinusitis with nasal polyps (CRSwNP) and asthma [30]. Table 1 summarizes the main characteristics of the studies focusing on Anti-IgE compounds.

### 3.2. Anti-IL5

IL-5 promotes eosinophil development and survival, so IL-5 is a target in the intention to reduce blood and tissue eosinophil counts [17]. Chronic sinusitis presents pathology associated with eosinophils and interleukin-5 (IL-5) in Caucasian patients. Patients who have CRSwNP with elevations in serum and mucosal eosinophils tend to present more severe NP disease. Despite chronic corticosteroid use, blood and tissue eosinophils may remain elevated, being associated with increased disease severity and recurrence rates postsurgery [31].

Three monoclonal antibodies have been approved until now. Mepolizumab, reslizumab and benralizumab are human monoclonal (IgG1) antibodies targeting interleukin 5 (IL-5) or the IL-5 receptor α subunit on the surface of eosinophils and are administered subcutaneously or intravenously [17].

***Benralizumab*** is an IL-5 receptor alpha-directed cytolytic monoclonal antibody that depletes eosinophils via cytotoxicity [32]. Benralizumab significantly improved NP score (−0.9 ± 0.2, *p* = 0.004) versus placebo (−0.3 ± 0.3, *p* = 0.166) and sinus occupancy. The antibody was well tolerated and improved symptoms and sensation of smell for most of the 24 patients included in the study by Tversky, Lane and Azar [33]. Subsequent studies extended the safety and efficacy profile of benralizumab in cases with NP regardless of severity and severe asthma. Benralizumab improved CRS for patients with severe, eosinophilic asthma and NP. Improvements in total SNOT-22 score were observed early in the therapy with the monoclonal antibody and maintained over time along the period of study for patients with a high baseline SNOT-22 total score (>30). Approximately half of the patients with asthma and NP treated with benralizumab achieved clinical improvements in SNOT-22 score and multiple asthma parameters (exacerbations, HRQoL, lung function, asthma control) [32]. A phase 2, randomized, double-blinded, placebo-controlled study was conducted in Japan by Takabayashi in patients with eosinophilic chronic sinusitis. A total of 56 patients were enrolled (11 with placebo, 22 with one dose of 30 mg benralizumab, 23 with 30 mg benralizumab every 4 weeks). Nasal polyp scores were reduced in the benralizumab group compared with the placebo group over the entire study period, especially in patients with high levels of blood eosinophils [34]. The phase 3 study OSTRO enrolled patients with severe symptomatic CRSwNP, despite treatment with intranasal corticosteroids, a history of systemic corticosteroid (SCS) use and/or surgery for nasal polyps (NP). The study population comprised 413 randomized patients (207 in the benralizumab group and 206 in the placebo group). When added to standard-of-care therapy, benralizumab reduced NPS, decreased nasal blockage and reduced problems with sense of smell compared to placebo in patients with CRSwNP [35]. Seventeen outpatients with severe eosinophilic asthma and CRSwNP were treated at the Osaka Habikino Medical Center with benralizumab for one year. Four weeks after treatment initiation, a rapid therapeutic action was noticed [36]. Benralizumab improved asthma control, lung function and sinonasal quality of life in the case series of patients with severe eosinophilic asthma and CRSwNP. Also, a number of patients presented an important in polyp burden [37]. The same results were obtained in Italy [38,39,40,41].

***Mepolizumab*** prevents IL-5 from connecting to its receptor on eosinophils and thus selectively inhibits eosinophilic inflammation [42]. Claus Bachert conducted a randomized, double-blind, placebo-controlled trial that recruited adult patients aged from 18 to 70 years with recurrent nasal polyposis requiring surgical intervention. A total of 750 mg of intravenous mepolizumab or placebo every 4 weeks for a total of six doses was administered in addition to daily topical corticosteroid treatment. The severity of nasal polyposis, VAS score, endoscopic nasal polyp score and sinonasal outcome were significantly improved with an important reduction in the need for surgery [43]. SYNAPSE is a randomized, double-blind, placebo-controlled, parallel-group, phase 3 trial that has been conducted in 11 countries (mostly in hospitals) including patients aged 18 years or older with recurrent, refractory, severe, bilateral nasal polyposis eligible for nasal surgery, despite standard therapy. Total endoscopic nasal polyp score significantly improved at week 52 from baseline in the arm with mepolizumab versus the placebo arm. Nasal obstruction VAS score significantly improved during weeks 49–52 [44]. The SYNAPSE trial showed that mepolizumab reduced the risk of recurrent sinus surgery in patients with advanced CRSwNP [38]. Mepolizumab reduced polyp size and nasal obstruction in chronic rhinosinusitis with NP regardless of the presence of comorbid asthma or AERD, according with the SYNAPSE trial [45]. Severe asthmatic patients with comorbid CRSwNP, treated with mepolizumab, were evaluated in a multicentric retrospective nonprofit observational study conducted in Italy. The authors evaluated SNOT-22 score, NP score and blood eosinophil count (and other CRS-specific variables) at baseline and after 12 months. All three variables investigated presented statistically significant reductions [46]. A significant reduction in SNOT-22, a decrease in TENPS (total endoscopic nasal polyp score), blood eosinophils and mean prednisone intake and improvement in %FEV1 and ACT were recorded in 44 severe eosinophilic asthma patients with CRSwNP treated with mepolizumab (100 mg q4w) for 1 year [47]. Treatment with mepolizumab reduced symptoms, polyp scores, blood eosinophils and systemic corticosteroid use in a single-center retrospective observational study in 55 patients [48].

***Reslizumab***—a phase 1, single-dose, randomized, double-blind, placebo-controlled, three-arm, parallel-group, two-center safety study of reslizumab in patients with nasal polyps was conducted by Gevaert. A total of 24 cases with massive bilateral nasal polyps were admitted into the study. A single injection of anti-IL-5 reslizumab reduced the size of nasal polyps in half of the patients, and the response to anti-IL-5 treatment was predicted using nasal IL-5 levels [49]. Table 2 summarizes the main characteristics of the studies about Anti-IL5 compounds.

### 3.3. Anti-IL4/IL13

***Dupilumab*** connects specifically to IL-4Rα, disrupting the activity of both IL-4 and IL-13 [50].

Eotaxin-3, total IgE, eosinophilic cationic protein, eotaxin-2 (*p* = 0.008), pulmonary and activation-regulated chemokine and IL-13 as biomarkers of type 2 inflammation in nasal secretions were reduced in CRSwNP patients receiving 300 mg dupilumab or placebo weekly for 16 weeks [51]. The addition of dupilumab (600 mg loading dose, then 300 mg once weekly for 15 weeks or matched placebo) reduced disease severity and significantly improved HRQoL based on SNOT-22, SF-36 and EQ-5D VAS scores in adults with CRSwNP refractory to INCS used alone to treat the pathology [52]. Two multinational, multicenter, randomized, double-blind, placebo-controlled, parallel-group studies (LIBERTY NP SINUS-24 and LIBERTY NP SINUS-52) assessed dupilumab added to standard treatment in adults with severe CRSwNP. Eligible patients for the study were people who were 18 years or older with bilateral CRSwNP and symptoms despite intranasal corticosteroid use, systemic corticosteroids received in the preceding 2 years or having had sinonasal surgery in the past years. In the SINUS-24 study, patients were assigned to receive 300 mg dupilumab or placebo every 2 weeks for 24 weeks, and in SINUS-52, they received 300 mg dupilumab every 2 weeks for 52 weeks and dupilumab every 2 weeks for 24 weeks and then every 4 weeks for the remaining 28 weeks or placebo every 2 weeks for 52 weeks. Dupilumab reduced sinus opacification, polyp size and the severity of clinical symptoms and was well tolerated [53]. Chuang and colleagues, based on these two studies, concluded that dupilumab-treated patients with CRSwNP compared with those with placebo arms demonstrated clinically important improvements in patient-reported sinonasal symptoms and objective outcome (NC, LoS, TSS, UPSIT, NPS and LMK-CT) [54]. A total of 60 adults with CRSwNP received weekly subcutaneous 300 mg dupilumab or placebo and daily mometasone furoate nasal spray in a 16-week randomized, double-blind, placebo-controlled, parallel-group study. In patients treated with dupilumab, a significant improved sinus opacification was noticed, measured via LMK (Zinreich-modified Lund–Mackay) scoring in all individual sinuses vs. placebo and correlated with SNOT22 smell/taste [55]. Rapid, significant and clinically meaningful improvements for nasal polyp score (NPS), nasal congestion (NC) score and sinus Lund–Mackay CT (LMK-CT) scores in 45 patients with CRSwNP in Japan in a 24-week study were provided via dupilumab [56]. Desrosiers showed that dupilumab lowers the number of patients undergoing sinonasal surgery and the use of systemic corticosteroids [57]. Dupilumab was an effective treatment for patients with severe CRSwNP regardless of prior sinus surgeries. Dupilumab reduced SCS use and/or the need for surgery. Dupilumab treatment showed greater improvements in objective outcomes of CRSwNP in patients with a shorter period since last sinus surgery [58]. Treatment with dupilumab led to important clinical improvements across many aspects of disease-specific HRQoL in patients with severe CRSwNP [59] and reduced hyposmia [60].

Dupilumab was also studied in patients with concomitant asthma and chronic rhinosinusitis. The five-dimension EuroQoL questionnaire (EQ-5D), visual analog scale (VAS) and 36-item Short-Form Health Survey (SF-36) were used by Bachert in a double-blind, placebo-controlled study that included a 4-week run-in and 16-week blinded treatment period to assess the effect of dupilumab on HRQoL in patients with CRSwNP with comorbid asthma. Patients considered eligible for the study were aged 18 to 65 years with bilateral NP and chronic symptoms of rhinosinusitis despite INCS treatment for more than 2 months and with more than two rhinosinusitis symptoms (nasal obstruction, nasal discharge, facial pain/pressure, reduction in/loss of smell). Improvements in clinical and patient-reported NP-specific outcomes and asthma-specific outcomes in patients with CRSwNP and comorbid asthma were demonstrated after dupilumab [29]. Patients with moderate-to-severe or OCS-dependent asthma with or without self-reported coexisting CRS-NP investigated in a long-term analysis presented a sustained reduction in exacerbations and improvements in lung function, asthma control and quality of life and an OCS dose reduction in baseline OCS-dependent patients after dupilumab [61]. A group of patients with CRSwNP and comorbid asthma received subcutaneously 300 mg dupilumab or placebo every 2 weeks supplementary to mometasone furoate nasal spray. Dupilumab improved many items concerning quality of life and medical points—the nasal polyp score, patient-reported nasal congestion score, Lund–Mackay computed tomography scan score, peak nasal inspiratory flow and 22-item Sino-Nasal Outcome Test score [62]. An amount of 200 mg/300 mg dupilumab reduced annualized severe exacerbation rates in 382 asthmatic patients with CRS and in 1520 patients without CRS [63]. Dupilumab was investigated in many countries in real-life situations. Hoffman et al. investigated 40 patients with uncontrolled severe chronic rhinosinusitis with nasal polyps in a single-center, retrospective single-arm longitudinal study. SNOT-22 and nasal polyp score (NPS) were improved [64]. An amount of 300 mg of dupilumab administered at home via a prefilled autoinjector every two weeks, based on indications set by the Italian Medicines Agency, was effective in reducing local nasal eosinophilic infiltrate, the need for surgery and/or oral corticosteroids, size of polyps and symptoms of disease. The improvements were observed as soon as 15 days from the beginning of the treatment and maintained in the next 12 months [65]. Patients affected by severe uncontrolled CRSwNP according to EPOS 2020 and under observation at the Otolaryngology Section of the University Hospital of Padova were included in an observational study in a real-life setting. A reduction in the local nasal inflammation, as measured via the numbers of eosinophil and neutrophil, was observed at 1, 6 and 12 months after the start of the treatment. Also, an important correlation between the reported sense of smell, measured via VAS, and the smell function, evaluated using SSIT, was observed at baseline as well as at T0, T3 and T6 [66]. The Otorhinolaryngology Unit of the University Hospital of Messina carried out an observational cohort study considering all patients treated with dupilumab. A total of 63 patients were included in the study. The Sino-Nasal Outcome Test 22 (SNOT-22) and nasal polyps score (NPS) were shown to present significant reductions at the 6th and 12th months compared to baseline values [67,68]. Table 3 summarizes the characteristics of main studies about the Anti-IL4/IL13 compounds.

## 4. Comparative Studies between Biologics

In the last years, authors from different countries have studied the differences between biologics and between surgery and biologics. Olfaction (anosmia, hyposmia) was improved in a real-world study of patients with CRSwNP with four monoclonal antibodies (omalizumab [35.8%], mepolizumab [35.4%], reslizumab [35.7%] and benralizumab [39.1%]), with no differences between the groups. The authors performed a multicenter, noninterventional, retrospective, observational, real-life study in nine hospitals belonging to the Spanish Asthma Network. The study population was 545 patients aged ≥18 years diagnosed with severe asthma and CRSwNP (diagnosed based on the presence of sinonasal symptoms and nasal endoscopy and/or CT findings). Approximately 40% of patients reported a subjective improvement in olfaction (with nonsignificant differences between biologic drugs) [69]. Soyka et al. collected data from 2014 to 2020 from forty-eight treatments in 29 patients aged between 27 and 70 years. The best success was shown using mepolizumab (78.9%), omalizumab (50%) and benralizumab (50%). A correlation between biomarkers and treatment success could not be found [70]. Otten, van der Lans, Fokkens and their colleagues studied ninety-four patients who were switched from one biologic to another for their treatment of CRSwNP and asthma. They concluded that dupilumab should be the first choice when switching biologic agents, based on the fact that most patients that failed with omalizumab and/or an anti-IL-5 treatment were well controlled on dupilumab. It was ineffective to switch to a second anti-IL5 treatment if the first one was not successful [71]. Ulrike Förster-Ruhrmann et al. conducted a monocentric study that recruited patients from 2012 to 2021 with SA and CRSwNP. One hundred and fifteen adult patients with SA and CRSwNP receiving one of the four biologics (mepolizumab 31; benralizumab 27; dupilumab 27; omalizumab 30) were included. Asthma Control Test, FEV1%, VAS, and total and nasal RSOM-31 subscores improved in all treatment groups. The most significant differences in pre/postscores were observed in the patients receiving dupilumab AND with no significant changes in the VAS scores, loss of smell in the benralizumab group and postnasal drip in the mepolizumab group [72]. Dupilumab exhibits the best efficacy and safety for the treatment of CRSwNP according to Luo Zhang et al., who evaluated seven RCTs (Bachert 2017, OSTRO, POLYP 1, POLYP 2, SINUS-24, SINUS-52 and SYNAPSE) involving 1913 patients and four biologics (benralizumab, dupilumab, mepolizumab and omalizumab [73]. ESS and dupilumab offered comparable improvement in smell identification at 24 weeks. ESS offered significantly greater reductions in polyp size compared to omalizumab, dupilumab and mepolizumab therapies in 111 patients suffering from CRSwNP [74]. ESS showed comparable improvement in quality of life and symptoms to omalizumab, mepolizumab and benralizumab. Dupilumab seemed to be more effective than ESS in selected items in a network meta-analysis [75]. Solano J and colleagues included 38 patients in an observational study of adults on anti-IL5 m-AB treatment: 19 patients received mepolizumab, 17 benralizumab and 2 patients reslizumab. There was a statistically significant difference in the ACT and SNOT-22 scores before and after mAb treatment with no significant differences between mAb groups [76]. On the other hand, a recent review analyzed from a critical point of view the methodology of research in this field. Borish et al. pointed out some aspects that need more attention in the future: an accurate and clinically meaningful characterization of baseline CRSwNP, different enrollment criteria for phase 3 clinical trials, differences in treatment populations, the need to use head-to-head comparisons, comparable patient populations and standardized outcome measures [77]. The Committee for Medicinal Products for Human Use (CHMP) of the European Medicine Agency approved Dupixent (dupilumab) as an add-on therapy with intranasal corticosteroids for the treatment of adults with severe CRSwNP for whom therapy with systemic corticosteroids and/or surgery does not provide adequate disease control (19.09.2019-EMA/CHMP/506821/2019), Xolair (omalizumab) as an add-on therapy with intranasal corticosteroids (INC) for the treatment of adults (18 years and above) with severe CRSwNP for whom therapy with INC does not provide adequate disease control (25.06.2020-EMA/339453/2020) and Nucala (mepolizumab) as an add-on therapy with intranasal corticosteroids for the treatment of adult patients with severe CRSwNP for whom therapy with systemic corticosteroids and/or surgery does not provide adequate disease control (16 September 2021-EMA/CHMP/2165/2021). Officially, in Europe, Fasenra (benralizumab) is indicated as an add-on maintenance treatment in adult patients with severe eosinophilic asthma inadequately controlled despite high-dose inhaled corticosteroids plus long-acting β-agonist (updated 21 September 2023), and Cinqaero (reslizumab) is indicated as an add-on therapy in adult patients with severe eosinophilic asthma inadequately controlled despite high-dose inhaled corticosteroids plus another medicinal product for maintenance treatment (updated 26 May 2023). The U.S. Food and Drug Administration approved Dupixent (dupilumab) to treat adults with nasal polyps (growths on the inner lining of the sinuses) accompanied by chronic rhinosinusitis (prolonged inflammation of the sinuses and nasal cavity), being the first treatment approved for inadequately controlled chronic rhinosinusitis with nasal polyps (26 June 2019), and Xolair for the add-on maintenance treatment of nasal polyps in adult patients 18 years of age and older with an inadequate response to nasal corticosteroids (November 2020). The FDA has approved the monoclonal antibody mepolizumab as a treatment for patients with chronic rhinosinusitis with nasal polyps (CRSwNP), being the first anti-IL-5 biologic to be approved for use in adult patients with CRSwNP in the United States (July 2021). The FDA has declined to grant approval to AstraZeneca’s Fasenra (benralizumab) to treat individuals with inadequately controlled chronic rhinosinusitis with nasal polyps (CRSwNP) (March 2022).

## 5. Future Clinical Directions

**Figure 2:**
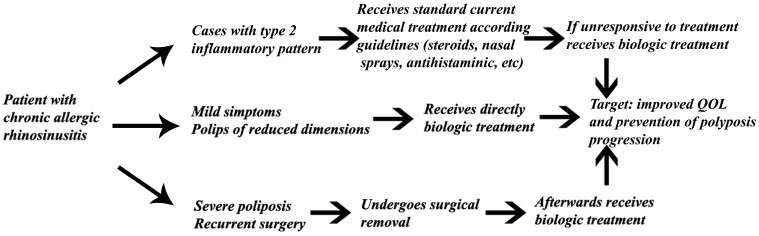
Future study design proposal for gathering data about the timing of biologic therapy initiation.

Currently, the guidelines for managing allergic chronic rhinosinusitis focus on symptomatic treatment and eventually surgical solutions like turbinate reduction with radiofrequency, coblation or endoscopic sinonasal surgery [78]. However, surgical management should be reserved for cases in which symptoms cannot be controlled [79]. Further evolution of the allergic chronic rhinosinusitis led to mucosal degeneration and the development of polyposis [80]. This raises the question of when biological treatment should be initiated. There are still debates whether biologic treatment could be initiated before surgical removal or after surgical removal of the polyps [81]. The main trend is focusing the research on preventing recurrence after surgical removal [82]. Among the factors responsible for recurrence after endoscopic sinus surgery are high levels of eosinophil cationic protein, high anti-double-stranded DNA IgG, high IL-5, high modified Lund–Mackay radiographic score and asthma [83]. There is still the question regarding the dimensions of the polyps and the level of polyposis that is still responding to the action of biological treatments [84]. The ultimate goal is the increase in the quality of life of the patient with chronic allergic rhinosinusitis [85]. EPOS2020 (The European Position Paper on Rhinosinusitis and Nasal Polyps—a long-standing initiative of the European Rhinologic Society in creating guidance for the management of patients with CRS) advised on the use of biologics in the treatment of CRSwNP. This update was written in collaboration with the European Forum for Research and Education in Allergy and Airway Diseases (EUFOREA), an international not-for-profit organization with the aim of preventing and improving the burden of chronic respiratory diseases. The authors considered that patients who have the proof of presence of bilateral polyps and have ESS in their medical history if they present evidence of type 2 inflammation, the need for systemic corticosteroids or contraindication of systemic steroids, significantly impaired quality of life, significant loss of smell and diagnosis of comorbid asthma, are suitable candidates for biologics. Five criteria were suggested to be evaluated for defining response: reduced nasal polyp size, a reduced need for systemic corticosteroids, improved quality of life and sense of smell and reduced impact of comorbidities at 16 and 48 weeks [86]. We believe that a personalized approach should be applied in any cases with initial symptomatic and surgical treatment, followed by administering biologic treatment for prevention of recurrence, Figure 2.

## 6. Conclusions

Biologics represents a new option in recent years in the treatment of chronic rhinosinusitis with nasal polyps. The present guidelines encourage the use of these compounds in selected patients. An important problem that must be resolved is the accessibility (logistic, financial) to this therapy compared to the surgical solutions in many countries, an aspect that is beyond the scope of this article. There is still a lack of head-to-head studies to provide evidence of the real-world effectiveness of different biologic drugs. EUFOREA guidelines may offer a solution for selected patients and increase their quality of life. In the future, those with chronic rhinosinusitis with nasal polyps may benefit from medical treatment with biologics and surgical solutions from case to case.

## Figures and Tables

**Table 1:** Anti-IgE—study characteristics.

Study	Year	Patients	Controls	Country	Age	Type of Study	Duration of Treatment	Prior Surgical Intervention
Pinto JM [21]	2010	7	7	USA	Adults (18–75)	Randomized, placebo-controlled double-blind study	24 weeks	Yes
Tajiri T [22]	2013	6	0	Japan	Adults	Prospective, uncontrolled study	16 weeks	No
Gevaert P [23]	2020	127	127	USA, Europe	Adults (18–75)	Two randomized phase 3 trials	24 weeks	
Kobayashi [24]	2021	25	0	Japan	Adults	Real-life study ECRS + Asthma	52 weeks	No
Bidder T [27]	2018	13	24 (surgery)	United Kingdom	Adults	Prospective study	16 weeks	Yes
Carceller M [28]	2020	23	0	Spain	Adults	Multicenter retrospective analysis	52 weeks	No
Haxel BR [26]	2022	21 omalizumab	49 dupilumab	Germany	Adults	Per protocol analysis—this real-world study		Yes
Tat T [29]	2022	17	0	Turkey	Adults	Real-life experience study	36 weeks	Yes
Zheng M [30]	2022	22	0	China	Adults	Real-life prospective study	24 weeks	No

**Table 2:** Anti-IL5—study characteristics.

Study	Year	Patients	Controls	Country	Age	Type of Study	Duration of Treatment	Prior Surgical Intervention
Tversky (benralizumab) [33]	2021	12	12	USA	Adults	Randomized double-blind placebo-controlled trial	20 weeks	Yes
Takabayashi (benralizumab) [34]	2021	46	11	Japan	Adults	A phase II, multicenter, randomized, placebo-controlled study	12 weeks	No
Matsuno O (benralizumab) [36]	2020	17	0	Japan	Adults	Retrospectively real-life, single-center study	52 weeks	No
Lombardo M (benralizumab) [38]	2020	10	0	Italy	Adults	Observational study	24 weeks	Yes
Chitguppi (benralizumab) [37]	2021	23	0	USA	Adults	Retrospective review	16 weeks	Yes
Nolasco S (benralizumab) [39]	2021	137	0	Italy	Adults	Multicenter observational study	24 weeks	Yes
Bachert C (benralizumab) [35]	2021	207	206	Europe	Adults	Randomized, placebo-controlled trial	12 weeks	Yes
Cavaliere C (benralizumab) [40]	2022	11	10	Italy	Adults	Retrospective study	52 weeks	Yes
Santomasi C (benralizumab) [41]	2023	17	0	Italy	Adults	Bse	52 weeks	No
Bachert C (mepolizumab) [43]	2017	54	51	Europe	Adults (18–79 years)	Randomized, double-blind, placebo-controlled trial	25 weeks	
Joseph K Han (mepolizumab) [44]	2021	206	201	Europe, North America	Adults	Randomized, double-blind, placebo-controlled, phase 3 trial	52 weeks	
Detoraki A (mepolizumab) [47]	2021	44	0	Italy	Adults	Prospective observational study	48 weeks	Yes
Bachert C (mepolizumab) [45]	2022	206	201	Europe, North America	Adults	Phase 3, randomized, double-blind, placebo-controlled, parallel-group study	52 weeks	
Gallo S (mepolizumab) [46]	2022	43	0	Italy	Adults	Multicentric retrospective no-profit observational study on severe asthmatic patients, treated with mepolizumab, and comorbid CRSwNP	52 weeks	
Dominguez-Sosa (mepolizumab) [48]	2023	55	0	Spain	Adults	Single-center retrospective observational study	24 weeks	Yes
Gevaert P (reslizumab) [49]	2006	24		Belgium	Adults	Double-blind, placebo-controlled, randomized, two-center safety and pharmacokinetic study	8 weeks	

**Table 3:** Anti-IL4/IL13—study characteristics.

Study	Year	Patients	Controls	Country	Age	Type of Study	Duration of Treatment	Prior Surgical Intervention
Jonstam K [51]	2019	30	30	USA, Europe	Adults	Randomized, double-blind, placebo-controlled, parallel-group study	16 weeks	
Bachert C [52]	2019	16	19	Europe	Adults (18–65)	Randomized, double-blind, placebo-controlled study	20 weeks	
Bachert C [53]	2019	143 (SINUS-24) 150 (SINUS-52)	133 (SINUS-24) 153 (SINUS-52)	14 countries	Adults	Two multinational, multicenter, randomized, double-blind, placebo-controlled, parallel-group studies	24 weeks SINUS 24 52 weeks SINUS 52	
Bachert C [55]	2020	30	30	USA, Europe	Adults (18–65)	Randomized, double-blind, placebo-controlled, parallel-group study	16 weeks	
Fujieda S [56]	2021	33	16	Japan	Adults	Phase 3, international, multicenter, randomized, placebo-controlled, double-blind SINUS-52 study	52 weeks	
Berger P [61]	2023	1101	611	Europe, USA	Adults	TRAVERSE (NCT02134028) was an OLE that enrolled patients who had completed a previous phase 2 or 3 dupilumab asthma study	96 weeks	
Hoffman A [64]	2022	40	0	Germany	Adults	Single-center, retrospective single-arm longitudinal study	53 weeks	Yes
De Corso E [65]	2023	57	0	Italy	Adults	Monocentric observational study in a real-life setting	48 weeks	Yes
Ottaviano G [66]	2023	47	0	Italy	Adults	Observational study in a real-life setting	48 weeks	Yes
Galletti C [67]	2023	63	0	Italy	Adults	Observational cohort study	48 weeks	Yes
Albrecht T [68]	2023	68	0	Germany	Adults	Prospective study	48 weeks	Yes

## Data Availability

Available on request from the corresponding authors.
